# Supplementary material for: GABAergic inhibition in human hMT+ predicts visuo-spatial intelligence mediated through the frontal cortex
Source: eLife. 2024 Oct 1;13:RP97545. doi: 10.7554/eLife.97545 (PMC11444681; doi:10.7554/eLife.97545)
Supplement: Supplementary file 1. [file elife-97545-supp1.docx]

**Supplementary File 1. FC of voxels showing significant correlation with BDT scores across subjects in whole brain.**

| FC number | Connected regions | BA | Size | Peak coordinate | *r* | *P* |
| --- | --- | --- | --- | --- | --- | --- |
|  |  |  |  | MNI (*x, y, z*) |  |  |
| 1 | Frontal_Med_OrbR | 11 | 44 | (2,43.5, -12) | -0.58 | 0.0009 |
| 2 | Frontal_Inf_Oper_R | 45 | 49 | (43.5,16.5,6) | 0.64 | 0.0002 |
| 3 | Precentral_L | 6 | 46 | (-49.5, -1.5,34.5) | 0.59 | 0.0007 |
| 4 | Precentral_L | 6 | 237 | (-24, -18,66) | 0.68 | 0.0001 |
| 5 | Precentral_R | 6 | 80 | (31, -25,72) | 0.67 | 0.0001 |
| 6 | Frontal_Mid_L | 10 | 82 | (-33,48,12) | 0.62 | 0.0003 |
| 7 | Insula_L | 47 | 124 | (-33,15, -9) | 0.64 | 0.0002 |
| 8 | Insula_L | 13 | 107 | (-31.5,9,10.5) | 0.63 | 0.0002 |
| 9 | Insula_L | 13 | 44 | (-42, -10.5,7.5) | 0.64 | 0.0002 |
| 10 | Frontal_Inf_Oper_R | 44 | 49 | (51,7.5,21) | 0.59 | 0.0007 |
| 11 | Frontal_Inf_Oper_R | 46 | 96 | (49.5,16.5,28.5) | -0.62 | 0.0003 |
| 12 | Frontal_Mid_R | 10 | 102 | (31.5,36,30) | 0.59 | 0.0009 |
| 13 | Paracentral_Lobule_L | 6 | 46 | (-15,21,51) | -0.64 | 0.0002 |
| 14 | Supp_Motor_Area_L | 6 | 48 | (-10.5,6,54) | 0.63 | 0.0003 |
| 15 | Frontal_Mid_L | 6 | 119 | (-24,4.5,55.5) | -0.67 | 0.0001 |
| 16 | Frontal_Sup_R | 6 | 136 | (29, -9,65) | 0.57 | 0.0014 |
| 17 | Frontal_Sup_MedialR | 9 | 90 | (8,51,43) | -0.59 | 0.0009 |
| 18 | Frontal_Mid_R | 9 | 108 | (50,19,41) | -0.67 | 0.0001 |
| 19 | Occipital_Mid_L | 19 | 47 | (-43, -83,9) | -0.64 | 0.0002 |
| 20 | Occipital_Mid_L | 37 | 45 | (-40.5, -63,4.5) | -0.62 | 0.0004 |
| 21 | Temporal_Mid_R | 39 | 56 | (45, -57,4.5) | -0.71 | 0.0000 |
| 22 | Temporal_Mid_L | 39 | 54 | (-45, -48,12) | -0.55 | 0.0021 |
| 23 | Temporal_Mid_L | 40 | 102 | (-48, -55.5,16.5) | -0.65 | 0.0001 |
| 24 | Temporal_Mid_R | 21 | 105 | (64, -2, -19) | -0.61 | 0.0004 |
| 25 | Temporal_Sup_R | 22 | 228 | (65, -42,11) | -0.6 | 0.0006 |
| 26 | Precuneus_L | 30 | 155 | (1.5, -51,16.5) | -0.64 | 0.0002 |
| 27 | Cingulum_Ant_R | 32 | 41 | (9,15,27) | 0.68 | 0.0000 |
| 28 | Cingulum_Mid_R | 31 | 73 | (15, -46.5,36) | -0.63 | 0.0002 |
| 29 | Cingulum_Mid_L | 23 | 80 | (-3, -15,30) | 0.63 | 0.0003 |
| 30 | Lingual_R | 18 | 46 | (21, -93, -16) | 0.58 | 0.0009 |
| 31 | Parietal_Sup_L | 7 | 54 | (-19.5, -63,55.5) | 0.66 | 0.0001 |
| 32 | Parietal_Sup_L | 7 | 48 | (-23, -71,58) | 0.63 | 0.0003 |
| 33 | Postcentral_L | 40 | 41 | (-31.5, -39,58.5) | 0.68 | 0.0001 |
| 34 | Postcentral_L | 40 | 40 | (-48, -36,58) | 0.71 | 0.0000 |
| 35  36  37 | Thalamus_L  Parietal_Sup_L  Vermis_10 | Wm  5  - | 108  37  37 | (-24, -31.5,12)  (-37, -48,63)  (-3, -43, -37) | 0.64 | 0.0002 |
|  |  |  |  |  | 0.59 | 0.0008 |
|  |  |  |  |  | -0.68 | 0.0001 |
| 38 | - | - | 40 | (12, -19.5, -42) | -0.68 | 0.0000 |

Single voxel threshold *P* < 0.01 (t > 2.771 or t < -2.771), adjacent size ≥ 37 voxels (AlphaSim corrected).
