# Supplementary material for: GABAergic inhibition in human hMT+ predicts visuo-spatial intelligence mediated through the frontal cortex
Source: eLife. 2024 Oct 1;13:RP97545. doi: 10.7554/eLife.97545 (PMC11444681; doi:10.7554/eLife.97545)
Supplement: Supplementary file 2. [file elife-97545-supp2.docx]

**Supplementary File 2. FCs of voxels showing significant correlation with SI across subjects in frontal cortex.**

| FC number | Connected regions | BA | Size | Peak coordinate | *r* | *P* |
| --- | --- | --- | --- | --- | --- | --- |
|  |  |  |  | MNI (*x, y, z*) |  |  |
| 1 | Frontal_Inf_Oper_R | 46 | 80 | (48,15,28.5) | -0.65 | 0.0001 |
| 2 | Precentral_R | 4/6 | 106 | (33, -25.5,63) | 0.72 | 0.0000 |
| 3 | Precentral_L | 6 | 26 | (-30, -24,72) | 0.66 | 0.0001 |

Single voxel threshold *P* < 0.005 (t > 3.057 or t < -3.057), adjacent size ≥ 22 voxels (AlphaSim corrected).
