# Supplementary material for: GABAergic inhibition in human hMT+ predicts visuo-spatial intelligence mediated through the frontal cortex
Source: eLife. 2024 Oct 1;13:RP97545. doi: 10.7554/eLife.97545 (PMC11444681; doi:10.7554/eLife.97545)
Supplement: Supplementary file 3. [file elife-97545-supp3.docx]

**Supplementary File 3. FCs of voxels showing significant correlation with SI across subjects in whole Brain.**

| FC number | Connected regions | BA | Size | Peak coordinate | *r* | *P* |
| --- | --- | --- | --- | --- | --- | --- |
|  |  |  |  | MNI (*x, y, z*) |  |  |
| 1 | Cerebelum_Crus1_L | 18 | 70 | (-29, -85, -24) | 0.51 | 0.0052 |
| 2 | Cerebelum_6_R | 18 | 39 | (12, -85, -17) | 0.65 | 0.0002 |
| 3 | Calcarine_R | 18 | 70 | (15, -91.5,12) | 0.63 | 0.0003 |
| 4 | Frontal_Inf_Oper_R | 46 | 127 | (48,15,28.5) | -0.65 | 0.0001 |
| 5 | Precentral_R | 4/6 | 179 | (32, -24,70) | 0.71 | 0.0001 |
| 6 | Precentral_L | 6 | 59 | (-30, -23,72) | 0.66 | 0.0001 |
| 7 | - | - | 37 | (26, -37, -11) | -0.67 | 0.0001 |

Single voxel threshold *P* < 0.01 (t > 2.771 or t < -2.771), adjacent size ≥ 37 voxels (AlphaSim corrected).
