# Supplementary material for: GABAergic inhibition in human hMT+ predicts visuo-spatial intelligence mediated through the frontal cortex
Source: eLife. 2024 Oct 1;13:RP97545. doi: 10.7554/eLife.97545 (PMC11444681; doi:10.7554/eLife.97545)
Supplement: Supplementary file 4. [file elife-97545-supp4.docx]

**Supplementary File 4. Correlations between FC in** **Supplementary File 1 and GABA/Glu concentrations in hMT+**

| FC number | hMT+ GABA concentrations | | | hMT+ Glu concentrations | | |
| --- | --- | --- | --- | --- | --- | --- |
|  | *r* | *P* | ***FDR*** | *r* | *P* | ***FDR*** |
| 1 | -0.13 | 0.49 | 0.55 | -0.08 | 0.68 | 0.84 |
| 2 | 0.17 | 0.38 | 0.49 | 0.05 | 0.82 | 0.88 |
| 3 | 0.39 | 0.04 | 0.22 | 0.22 | 0.24 | 0.69 |
| 4 | 0.41 | 0.03 | 0.19 | 0.24 | 0.21 | 0.69 |
| 5 | **0.69** | **0.0001** | **0.0038^**^** | 0.47 | 0.01 | 0.38 |
| 6 | 0.2 | 0.3 | 0.41 | 0.1 | 0.6 | 0.81 |
| 7 | 0.26 | 0.17 | 0.34 | 0.21 | 0.28 | 0.69 |
| 8 | 0.22 | 0.24 | 0.35 | 0.25 | 0.19 | 0.69 |
| 9 | 0.23 | 0.23 | 0.35 | 0.02 | 0.9 | 0.92 |
| 10 | -0.13 | 0.49 | 0.55 | -0.16 | 0.41 | 0.70 |
| 11 | **-0.56** | **0.0018** | **0.03^*^** | -0.22 | 0.25 | 0.69 |
| 12 | 0.2 | 0.3 | 0.41 | 0.05 | 0.81 | 0.88 |
| 13 | -0.35 | 0.06 | 0.25 | 0.07 | 0.73 | 0.84 |
| 14 | 0.39 | 0.03 | 0.19 | 0.31 | 0.1 | 0.69 |
| 15 | 0.006 | 0.98 | 0.98 | 0.14 | 0.46 | 0.70 |
| 16 | 0.26 | 0.18 | 0.34 | 0.16 | 0.4 | 0.70 |
| 17 | 0.15 | 0.42 | 0.51 | 0.04 | 0.83 | 0.88 |
| 18 | -0.4 | 0.03 | 0.19 | -0.21 | 0.27 | 0.69 |
| 19 | -0.37 | 0.05 | 0.24 | -0.15 | 0.44 | 0.70 |
| 20 | -0.16 | 0.39 | 0.49 | -0.19 | 0.33 | 0.70 |
| 21 | -0.31 | 0.1 | 0.28 | -0.29 | 0.13 | 0.69 |
| 22 | -0.22 | 0.24 | 0.35 | -0.07 | 0.72 | 0.84 |
| 23 | -0.1 | 0.6 | 0.65 | -0.12 | 0.53 | 0.77 |
| 24 | -0.09 | 0.64 | 0.68 | -0.09 | 0.63 | 0.83 |
| 25 | -0.3 | 0.11 | 0.28 | -0.14 | 0.46 | 0.70 |
| 26 | -0.08 | 0.66 | 0.68 | -0.08 | 0.69 | 0.84 |
| 27 | 0.28 | 0.13 | 0.29 | 0.1 | 0.59 | 0.81 |
| 28 | -0.27 | 0.16 | 0.34 | -0.2 | 0.29 | 0.69 |
| 29 | 0.33 | 0.08 | 0.28 | 0.25 | 0.19 | 0.69 |
| 30 | 0.24 | 0.21 | 0.35 | 0.19 | 0.32 | 0.70 |
| 31 | 0.24 | 0.22 | 0.35 | 0.36 | 0.05 | 0.69 |
| 32 | 0.23 | 0.24 | 0.35 | 0.23 | 0.23 | 0.69 |
| 33 | 0.44 | 0.02 | 0.19 | 0.18 | 0.35 | 0.70 |
| 34 | 0.32 | 0.09 | 0.28 | 0.002 | 0.99 | 0.99 |
| 35 | 0.32 | 0.09 | 0.28 | 0.31 | 0.1 | 0.69 |
| 36 | 0.30 | 0.11 | 0.28 | 0.22 | 0.25 | 0.69 |
| 37 | -0.29 | 0.13 | 0.29 | -0.23 | 0.23 | 0.69 |
| 38 | -0.13 | 0.48 | 0.55 | -0,15 | 0.42 | 0.70 |

Bold font indicates the significant correlations survived from multi correlation correction.
