# Supplementary material for: GABAergic inhibition in human hMT+ predicts visuo-spatial intelligence mediated through the frontal cortex
Source: eLife. 2024 Oct 1;13:RP97545. doi: 10.7554/eLife.97545 (PMC11444681; doi:10.7554/eLife.97545)
Supplement: Supplementary file 5. [file elife-97545-supp5.docx]

**Supplementary File 5. Correlations between FCs in Supplementary File 2 and GABA/Glu concentrations in hMT+.**

| FC number | hMT+ GABA concentrations | | | hMT+ Glu concentrations | | |
| --- | --- | --- | --- | --- | --- | --- |
|  | *r* | *P* | ***FDR*** | *r* | *P* | ***FDR*** |
| 1 | **-0.56** | **0.0017** | **0.0017**** | -0.21 | 0.27 | 0.27 |
| 2 | **0.70** | **0.0001** | **0.0003***** | **0.49** | **0.007** | **0.021*** |
| 3 | **0.65** | **0.0001** | **0.0002**** | **0.40** | **0.03** | **0.045*** |

^*:^ *P_FDR_* < 0.05; ^**:^ *P_FDR_* < 0.01; ^***:^ *P_FDR_* < 0.001; Bold font indicates the significant correlations survived from multi correlation correction.
