# Supplementary material for: GABAergic inhibition in human hMT+ predicts visuo-spatial intelligence mediated through the frontal cortex
Source: eLife. 2024 Oct 1;13:RP97545. doi: 10.7554/eLife.97545 (PMC11444681; doi:10.7554/eLife.97545)
Supplement: Supplementary file 6. [file elife-97545-supp6.docx]

**Supplementary File 6. Correlations between FCs in Supplementary File 3 and GABA/Glu concentrations in hMT+.**

| FC number | hMT+ GABA concentrations | | | hMT+ Glu concentrations | | |
| --- | --- | --- | --- | --- | --- | --- |
|  | *r* | *P* | ***FDR*** | *r* | *P* | ***FDR*** |
| 1 | **0.37** | **0.049** | **0.049^*^** | 0.32 | 0.09 | >0.05 |
| 2 | **0.37** | **0.049** | **0.049*** | 0.41 | 0.025 | >0.05 |
| 3 | **0.48** | **0.008** | **0.01^*^** | 0.21 | 0.28 | >0.05 |
| 4 | **-0.58** | **0.001** | **0.002^**^** | -0.22 | 0.26 | >0.05 |
| 5 | **0.69** | **0.0001** | **0.0007** | 0.46 | 0.01 | >0.05 |
| 6 | **0.66** | **0.0001** | **0.0004^***^** | 0,39 | 0.04 | >0.05 |
| 7 | **-0.48** | **0.0077** | **0.01*** | -0.35 | 0.067 | >0.05 |

^*:^ *P_FDR_* < 0.05; ^**:^ *P_FDR_* < 0.01; ^***:^ *P_FDR_* < 0.001; Bold font indicates the significant correlations survived from multi correlation correction.
